# Empowering her guardians to nurture our Ocean’s future

**DOI:** 10.1007/s11160-021-09679-3

**Published:** 2021-08-27

**Authors:** Mibu Fischer, Kimberley Maxwell, Nuunoq, Halfdan Pedersen, Dean Greeno, Nang Jingwas, Jamie Graham Blair, Sutej Hugu, Tero Mustonen, Eero Murtomäki, Kaisu Mustonen

**Affiliations:** 1 CSIRO Oceans and Atmosphere, Brisbane, QLD Australia; 2 Centre for Marine Socioecology, Hobart, TAS Australia; 3 Te Kōtahi Research Institute, University of Waikato, Tauranga, New Zealand; 4 The Pisuna Project, Attu, Greenland; 5 Pikkoritta Consult, Aasiaat, Greenland; 6 College of Arts, Law and Education, University of Tasmania, Launceston, TAS Australia; 7 Hereditary Chief, Haida Nation, Canada; 8 Institute for Marine and Antarctic Studies, University of Tasmania, Hobart, TAS Australia; 9 Indigenous Taiwan Self-Determination Alliance (ITW-SDA), Namasia, Taiwan; 10 Snowchange Cooperative, Selkie, Finland

**Keywords:** Indigenous, Traditional people, First Nations, Traditional ecological knowledge, Colonisation, Climate change

## Abstract

Coastal Indigenous and Traditional communities are starting to see changes to their lives from climate change, whether this is from species range changes or displacement from land changes. For many of these communities, the ability to adequately adapt to these changes is limited by the governance structures they are required to live within, which differ from their customary practices and culture. In November 2019, a group of Indigenous and Traditional Peoples, attended the Future Seas 2030 workshop and discussed the consequences of climate change, the biggest barriers for their communities, and barriers for using traditional knowledge in order to contribute towards a more sustainable future that in the end will benefit all of earth’s people. The aim of this workshop was to highlight and give a voice to the various backgrounds and real-life situations impacting on some of the world’s Indigenous and Traditional communities whose connection with the oceans and coasts have been disrupted. This paper presents these issues of oppression, colonisation, language and agency, making it difficult for these groups to contribute to the current management of oceans and coasts, and asks scientists and practitioners in this space to be allies and enable the needed shift to earth’s guardians taking a leading role in nurturing her for our future.

## Positionality

All authors on this paper identify as Indigenous, First Nations or Traditional People from diverse cultural, gender, age, and biodiverse backgrounds. Whilst there are differences between us all, there are similarities in the worldviews that we have grown up with. We acknowledge the multiple terms used and preferred by numerous Indigenous groups worldwide, for the purposes of this paper, through consensus, the authors have decided to use the term Indigenous and Traditional Peoples’ throughout the paper to refer to Indigenous, Traditional Peoples, First Nations communities and other similar groups as a diverse collective. The capitalisation of the word Indigenous gives commonality to a diverse group of people who have been impacted by colonisation. When talking about specific case studies or groups it is preferred to use the known terminology for that group i.e. when we refer to Canadian Indigenous peoples, we may use First Nations, Inuit, and Metis. We also use the plural term Peoples to refer to a collective group of populations. The reference to the ocean as ‘her’ relates to conversations between authors, it reflects an old Karelian concept that the sea is female (see Tero Mustonen et al. 2021).

The authorship team is led by Mibu Fischer, Mibu is a young Noonucal, Ngugi, and Goenpul woman from Quandamooka Country in South East Queensland, Australia. She identifies as a saltwater woman and works as an early career Marine Ethnoecologist. She is supported by ten knowledgeable individuals which includes Kimberley Maxwell, who is of Whakatōhea, Te Whānau-a-Apanui, Ngāti Porou, Ngāti Tūwharetoa and Ngāitai ki Umupuia descent, in the North Island, NZ. Since 2004 she has worked for Māori communities in the fields of customary fisheries, aquaculture, benthic ecology, wastewater management and more recently in fisheries, marine management, and planning, having completed her PhD in Dec 2019. Nuunoq (Per Ole Fredriksen) is a Greenlandic male whose livelihood is living off the local sea and land area in the small village of Attu on the west coast of Greenland. He was supported to participate in this project by translator, Halfdan who is an elderly Greenlandic male who has studied abroad and returned to his native Aasiaat not far from Attu. Dean Greeno is a sea country trawlwoolway pakana man from lutruwita, Australia. He is an Artist, Aircraft Engineer, Builder, and father. Chief Nang Jingwas (Russ Jones) is a Hereditary Chief of the Haida Nation who lives in his ancestral community of Skidegate, Haida Gwaii on the west coast of Canada. Retired but continues with work related to local and international fisheries, marine planning, and shipping. Jam Graham Blair is a young trawlwoolway plangermaireener pakana based in nipaluna, lutruwita, Australia. He is a cultural practitioner with specialist skills in environmental science, Indigenous resistance, and Traditional Knowledge. Sutej Hugu is a Siraya from Tavokan, now based in Pongo no Tao, a tribal activist who works on Indigenous decolonisation and sustainable self-determination. There is also a small contingent from Finland led by Elder Eero Murtomäki, who is a hunter and a nature photographer from the village of Petsmo, Vaasa region, Finland. From the village of Selkie, North Karelia, Tero Mustonen, a fisherman and researcher, and Kaisu Mustonen who is a scholar and Head of Biodiversity Unit at Snowchange Cooperative.

The authorship team have provided their connections to their ancestral homelands and waters for context. It is important to note that whilst the focus is on oceans, the discussions that fed into the paper extended beyond ocean systems. There are many communities who have an integral relationship and understanding of oceans, but case studies may be best reflected in a range of habitats.

## Preamble

The University of Tasmania, which is home to the Future Seas project, lies only a short distance from the site of an infamous massacre of local Indigenous peoples. As described in this paper, Indigenous and Traditional People across the world have much to contribute to a better future for our oceans. But meaningful dialogue and engagement on ocean issues requires recognition of historic injustices as the first step towards a true reconciliation. Stories such as the one below reveals an uncomfortable history, retelling such stories is an opportunity to educate and heal past traumas. This story has historical value but is also a story of survival of the original people who cared for these lands, waterways, seas, and skies in a sustainable manner for thousands of years. These original people continue to be the guardians of their unique spiritual and cultural lands and seascapes.

Standing around the fire on a fresh spring morning at piyura kitina, lutruwita (Risdon Cove, Tasmania), surrounded by gums and the occasional playful squeal of a child from the nearby childcare centre, in peaceful silence was a group of people, varying in age and ethnicity. What followed that tranquil moment was an introduction to the brutal history of the country they were gathered upon (Fischer 2019, personal communication, 11 November). The local Mumirimina palawa (Tasmanian Aboriginal People of the Pittwater & Risdon area) were custodians of this place. Guardians of the river, forest, and hunting plains for thousands of generations, it was these people whom thanks and respect was offered to in this gathering. Caretakers of a complex system of lore which had kept them in harmony with land, sea, and sky for millennia, only to be disrupted by the arrival of Lieutenant John Bowen and the first British colonialists. On this very spot on the 3^rd^ May 1804, while hunting for a large ceremonial gathering with the Big River palawa, the Mumirimina had circled a big mob of kangaroo and were driving them down towards the river. With cannons and muskets, the soldiers at Bowens camp opened fire on them all, killing men, women, and children who were armed with nothing but waddies.^1^ Many bodies of the deceased were packed into barrels of lime to remove the flesh and then shipped off to Sydney for scientific interest. The first child to be forcibly removed from his parents was stolen that day, christened, and given the new (and only recorded name) Robert Hobart May (Tasmanian Aboriginal Centre 2020). This began a legacy of forced child removal, violence, and dispossession that the palawa still feel and navigate today. But it is not only the palawa who felt this loss, but the land and waterways of the area too. You see at the time of writing this, Australia has recorded only two marine extinctions, both of which have occurred in the Derwent Estuary, the river that these Mumirimina palawa were responsible for caring for. Had their sovereignty been recognised, their rights to exist freely and uncolonized retained, the river would have remained healthy and these extinctions (and the many other forms of environmental damage witnessed in the Estuary) would most likely not have occurred (Graham Blair 2019, personal communication, 15 November).

The colonial settlements of Tasmania are built upon events and massacre sites similar to this place. This extends to both the academic and industrial institutions of the island, and indeed the accumulated knowledge and understanding of the island. Even though piyura kitana has since been returned to the palawa community, the pain and loss of this massacre, and the numerous other incidences of human rights abuses that occurred here, are still felt today, but seemingly only acknowledged and talked about by the Indigenous people (Graham Blair 2019, personal communication, 15 November).

Lutruwita’s brutal early interactions with the British, which nearly saw them wiped out within three decades, is not an isolated incident. Every continent has been invaded at some point in history, and the process of invasion has been honed and adapted to take supreme control over the Indigenous and Traditional communities, to the point where they are eliminated, or their history erased. Regardless of which foreign power (British, French, Spanish, Chinese, Portuguese…) was responsible for invading Indigenous and Traditional Peoples lands and waterways, the pathway to asserting foreign sovereignty has followed a repeated narrative. Separation of the generations is often a prominent part of colonisation and is a recognised form of genocide (Docker 2015; Krieken 1999; Schimmel 2005). By doing this, the transmission of intergenerational knowledge is interrupted, resulting in a loss of identity and key cultural responsibilities to land and water. Often during this cultural breakdown, new knowledge around language, religion, and social conventions is forced onto the younger generations to assimilate them to the invader’s philosophies and views. These forced assimilations also include massive changes to diets and subsequently overall health of communities, which to this day is still a prevalent issue (Griffiths et al. 2016; Sherwood 2013). Whilst this is not a comprehensive list of colonial impacts, it highlights the systematic and well-designed nature of invasion and conquest with widespread and parallel impacts on Indigenous and Traditional Peoples’ cultures, cosmologies and ecologies. The shared history of colonisation that many Indigenous and Traditional Peoples around the globe remember and live with is what brings them together in solidarity. Due to the nature of elimination, the domination of Indigenous and Traditional Peoples has resulted in thousands of disenfranchised communities, affecting over 5000 different Indigenous and Traditional communities worldwide (Amnesty International 2020). This highlights the importance of decolonisation, not only for Indigenous people and their usurped way of life, but also for the systems and institutions who benefit from their existence and treatment.

Going back to that cold spring morning at piyura kitina, standing amongst that group of people was a small collective of Indigenous and Traditional Peoples from across the world. We are now connected through the Future Seas 2030 Project, and the joint experiences we shared during the November 2019, Future Seas 2030 workshop.

## Introduction

For millennia, Indigenous and Traditional Peoples have successfully utilised marine and coastal resources using traditional management practices, practices which are rooted within their epistemologies and ontologies (Kinnear 2007; Prosper et al. 2011). For many communities the ability to continue these practices, in order to survive, has been reduced due to colonial impacts. Where practices remain or are revitalised, it is through the sheer persistence of the community.

For many Indigenous and Traditional Peoples, we come from the lands, waterways, and seas, and as such the stewardship for maintaining a healthy country is synonymous with maintaining our own health (Ganesharajah 2009; Jarvis 2019; Kingsley et al. 2013). Management structure is often through informal processes, tied to individual and community customary rights, ceremonies, songs, taboos, and totems which result in ‘restrictions’ in marine resource use between clan groups, and serve to achieve sustainability of the shared resources. Whilst these long successful and sustainable stewardship practices are deeply important and have strong cultural links to Indigenous and Traditional communities, they are marginalised and excluded in colonised management processes resulting in degraded marine and coastal environments. Many communities are striving to continue their stewardship over their homelands and waters by partnering with broader management entities (Ban and Frid 2018; Ban et al. 2008; Diggon et al. 2019; Nursey-Bray and Jacobson 2014; Ross et al. 2009).

This paper presents one version of a vision for a fair ocean future for Indigenous and Traditional Peoples around the world. The authors explicitly recognise that we are providing our perspectives for a future scenario. We do not represent all Indigenous and Traditional Peoples and are certainly not trying to create a one world vision for Indigenous and Traditional Peoples perspectives on climate change or sustainability challenges, as they relate to our oceans, as this would be impossible for our group to address given the diversity of Indigenous and Traditional Peoples of the world, the different languages, histories, environments and needs. Thinking this way would be irresponsible of us. Whilst a fair future marine environment is a common goal for the Future Seas papers, this paper differs in that the Indigenous and Traditional communities of the authors have often been disadvantaged through numerous drivers, which have been identified as colonialism, globalisation and agency (ability to adapt to change).

We begin by describing where and how we started, for this paper and this purpose, and historically, as Indigenous and Traditional Peoples (i.e., systematically disposed of our lands, our waters, and our oceans). Following this is a brief overview of Indigenous worldviews, followed by limitations around language. We then journey around the globe to hear of varying Indigenous and Traditional Peoples’ accounts of life with oceans. It should be noted that with the fluid nature of environmental knowledge many communities have, some case studies do move away from an ocean focus, however there are still lessons that can be drawn from these experiences and applied in a marine context.

Most case study narratives talk about the implications of colonialism on their culture, and the resulting impacts of these attempted genocides. Indigenous and Traditional Peoples ability to contribute to a sustainable future for all is predominantly impacted by foreign sovereignty, coupled with reductionism of our beliefs and governance systems, and rights and access to resources (Jalata 2013). Whilst some Indigenous and Traditional Peoples have been able to continue to practice their culture relatively uninterrupted by colonial powers, many are at risk of a second wave of attempted genocide from human-induced climate change and globalisation. The discussion section of this paper explores the various ways in which our case study narratives demonstrate the impacts of a generalised Business as Usual approach to oceans, as well as examples of positive steps available for foreign powers, including settler states, and Indigenous and Traditional Peoples, to work together. Beyond these positive steps, more can be done to allow Indigenous and Traditional Peoples to be a part of the solution for a sustainable ocean future for all. We conclude with our opinions on how to increase Indigenous and Traditional Peoples participation in climate change studies, solutions, and research moving forward.

## Worldviews

**Fig. 1:**
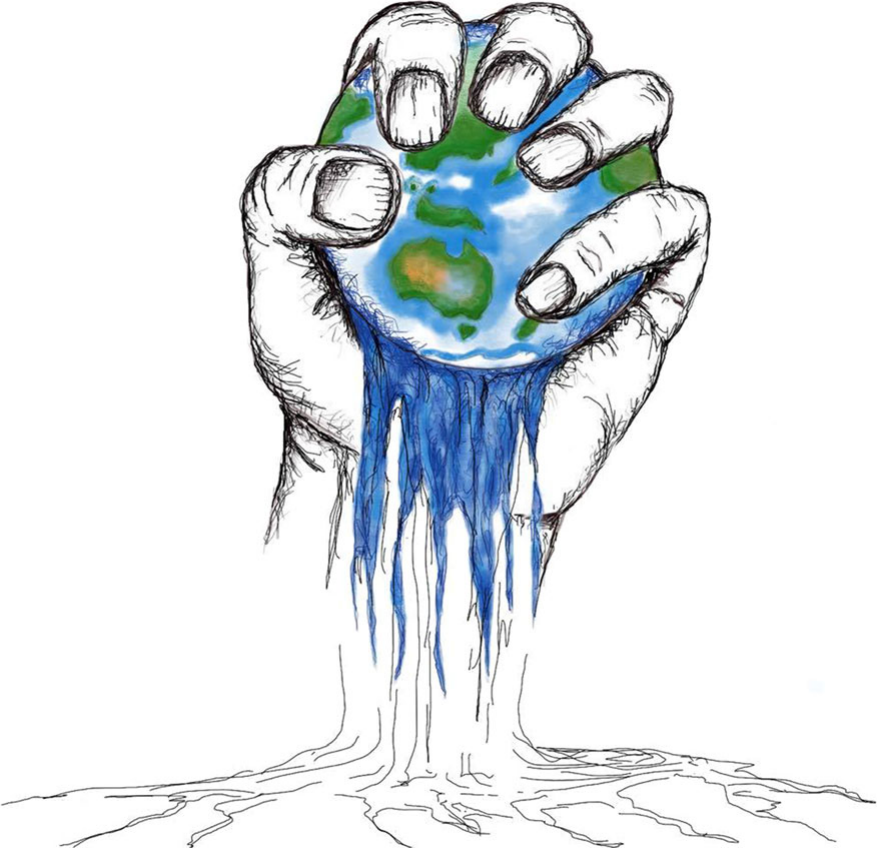
Western Worldviews (Business as Usual). Indigenous view of Westernisation destroying Mother Earth including the oceans through misuse of natural resources. The domination of westernisation globally was triggered through colonisation of many groups in the 16th, 17th, and 18th centuries, leading to the current unbalanced system. The authorship group discussed this concept throughout the Future Seas 2030 Workshop and those discussions were turned into this piece by artist and Pakana man Dean Greeno, for inclusion in this paper

According to the United Nations (UN), the upwards of 5000 different Indigenous and Traditional Peoples’ cultures contributes the majority of the 6000 world languages across 90 countries (UN DESA 2019; UNESCO 2018). There are approximately 370 million Indigenous and Traditional Peoples, making up nearly 5% of the world’s population (UNESCO 2018), there is a general understanding that seems to exist that more than half the world’s biodiversity, potentially more than 75% are to be found in areas under the auspices (in various forms) of Indigenous and Traditional Peoples (with marine areas yet to be included). Hence, these diverse ecosystems of Indigenous and Traditional Peoples territories contribute a great deal to the remaining 95% of the human population, although with an imbalance of power, economic disadvantage and more, for Indigenous and Traditional Peoples. The loss of natural environment and access to lands and waters, due to changing land use for the benefit of the foreign power is vast and of great concern to many Indigenous and Traditional Peoples, depicted in Fig. 1 (Cannon 1995; Kusiluka et al. 2011; Proce et al. 2006; Stavig 2000; Walls & Whitbeck 2012). The impacts from changes to the landscape that Indigenous and Traditional Peoples have felt, go beyond impacts of food security and enjoyment of place.

**Fig. 2:**
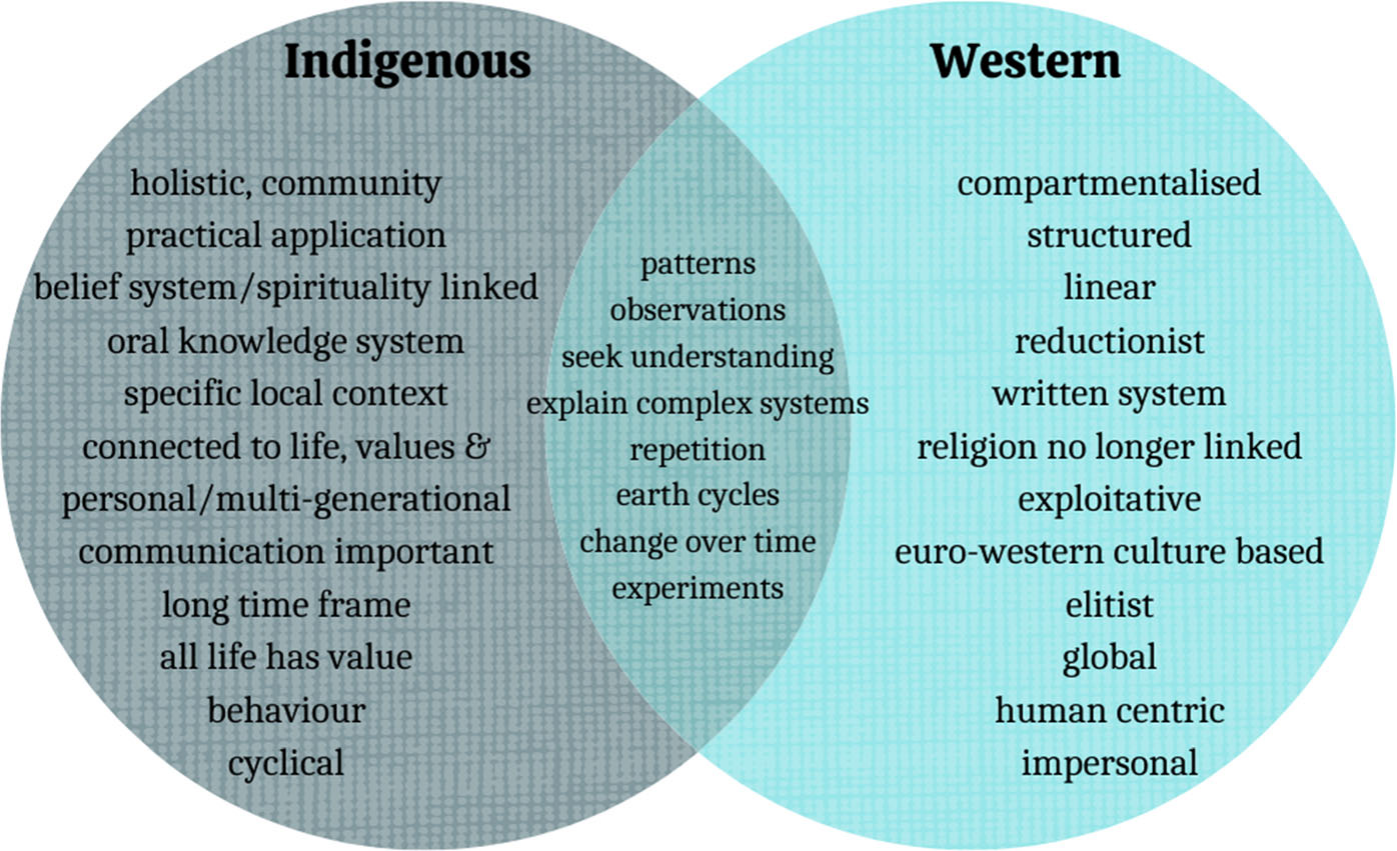
Comparisons of Indigenous and Western Worldviews. Whilst these systems are different, there are many similarities between the different versions of information sharing

In order to begin understanding Indigenous and Traditional Peoples perspectives there must be some understanding of our worldviews (Martin 2017). Common to Indigenous worldviews is recognising the interconnectedness and right to life that the environment and everything within it has, which differs from the reductionist Western mindset (Cajete 2000; Johnson 2012; Johnson et al. 2016; Le Grange 2007). These views can be called endemic or specific (Mustonen 2014). They are often non-global ways of thinking with inherent values themselves. This right to life is the main difference, where Indigenous and Traditional Peoples place humans within the system and are actively part of it, whilst the Western system places humans outside of the environment and views it as something to control (Fig. 2).

One can ask questions about the multiplicity of values that a landscape has for people. But these questions cannot readily be asked within an Aboriginal concept of country because country has its own life, its own imperatives, of which humans are only one aspect. It is not up to humans to take supreme control, or to define the ultimate values of country. (Rose, 1996 p.10)

**Fig. 3:**
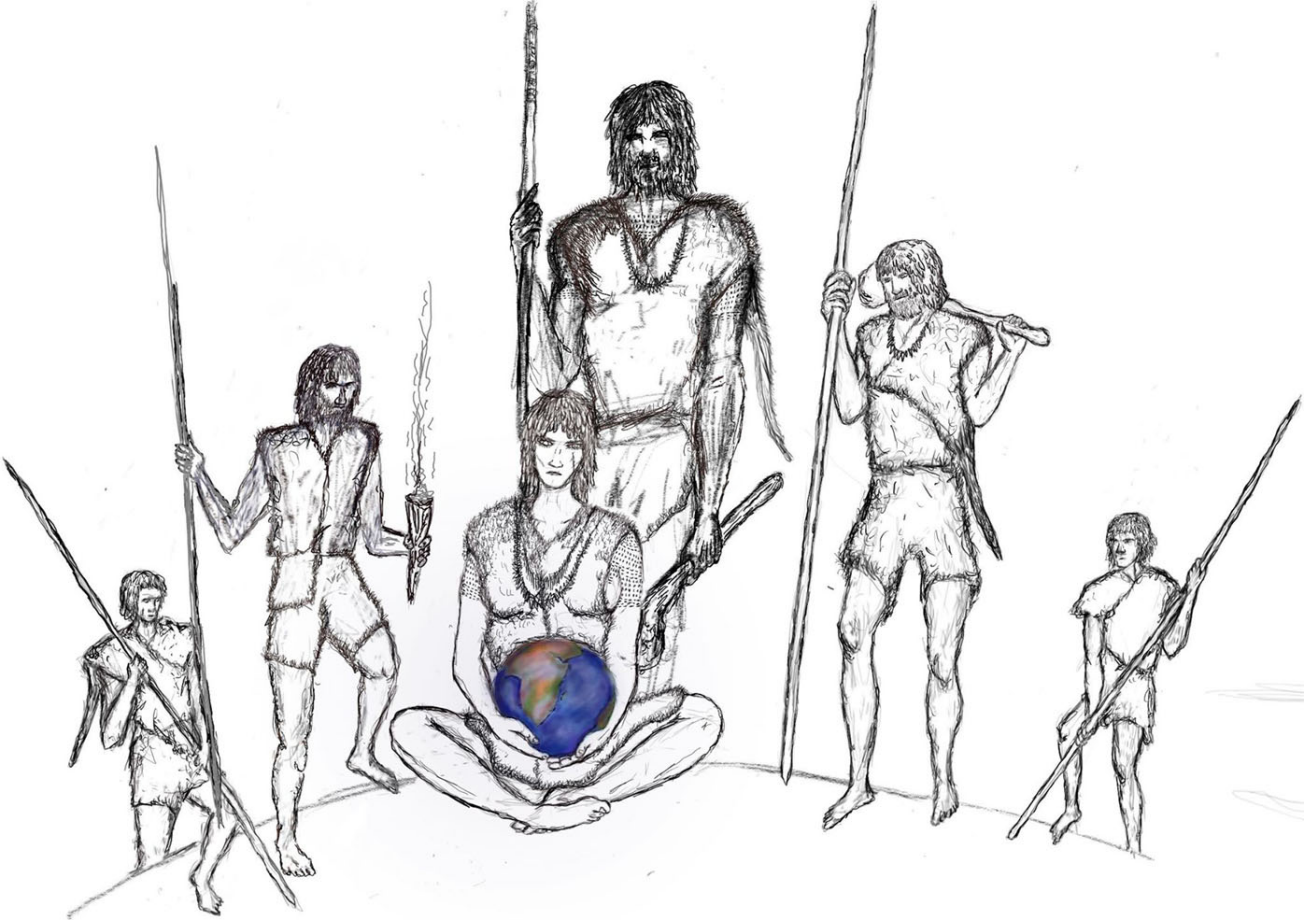
Indigenous Worldviews (Sustainable Future). Many discussions were had between the authorship team during the Future Seas 2030 Workshop. The topic of ancestral connection to our Mother oceans and earth was a constant thread throughout. The holistic, nurturing practices of Indigenous and Traditional Peoples were discussed and were interpreted and formed into this piece by artist and pakana man Dean Greeno specifically for this paper

Indigenous and Traditional Peoples have known the need to live a harmonious lifestyle since the beginning of time (Fig. 3), yet it is only now that we are being empowered to have a say in western governance systems and against the consumerist system that we have been dragged into. The myriad of climate change predictions has already begun impacting on Indigenous and Traditional Peoples’ lifestyles, including our culture (Mustonen et al. 2021, this issue). Therefore, listening to how Indigenous and Traditional Peoples can be part of the solution is one step towards tackling many mitigation and adaptation questions. Eero Murtomäki from Finland has defined this difference as one of harmony (natural system) and chaos (imposed colonial rule over nature). In a contemporary context where Indigenous and Western mindsets are being brought together several frameworks have been and continue to be developed, ‘Two-Eyed Seeing’ or Etuaptmumk in Mi’kmaw, is one of those principles that Mi’kmaw Elder Albert Marshall introduced into Integrative Science in 2004 (Bartlett et al. 2012).

## Language barriers

Before we continue, we must acknowledge that we are writing about Indigenous and Traditional Peoples ideals in English, which frequently is the language of science. A language which for some of us is foreign and for others was used as a tool to colonise our ancestors. It can continue to perpetuate harm to Indigenous and Traditional communities through the continued use of westernised names, especially those that reference past atrocities and at locations that have cultural significance i.e., Cape Grim, Tasmania. Language is a part of our knowledge systems, and we are not trying to disconnect the two by writing in English, but rather translate our perspectives and knowledge as Indigenous and Traditional Peoples on our own terms. The ability to translate the meaning behind traditional languages into a western context can be difficult in some instances, as the meaning and emphasis on particular words can be lost (Beard 2017), the importance can be captured in the quote from Decolonising the Mind, by famous African writer wa Thiong’o (1986).

Language carries culture, and culture carries, particularly through orature and literature, the entire body of values by which we come to perceive ourselves and our place in the world… Language is thus inseparable from ourselves as a community of human beings with a specific form and character, a specific history, a specific relationship to the world. (Thiong’o 1986, p.16)

In addition to difficulties in translation between languages, there is also tension between the two styles of knowledge sharing and accumulation reflected in Fig. 2 (the oral based knowledge system common to many Indigenous worldviews, in comparison to the written style system in the West). The more globally dominate system of the written word struggles to understand the validity of orally disseminated knowledge and continues to question the validity of traditional knowledge (Beard 2017; Tuhiwai Smith 2012). The desire for researchers and practitioners to request written information from traditional knowledge is problematic, for many reasons including misinterpretation or use of knowledge and historic literature written from a coloniser’s perspective (Russell 2005; Tuhiwai Smith 2012).

Two thousand and nineteen was the International Year of Indigenous Languages and was a catalyst for many nations to start to think about the benefits received from Indigenous and Traditional Peoples’ languages. Unfortunately, it was not enough to reduce the continued loss of languages that is occurring, with the UN stating 4 in 10 Indigenous languages are close to disappearing (UN News 2019).

Linda Tuhiwai Smith notes in her book, *Decolonising Methodologies,* the number of Indigenous communities who have stated ‘we are the most researched people in the world’ (Tuhiwai Smith 2012). As she discusses, it isn’t the truth of the statement, it’s the meaning behind it, Indigenous and Traditional Peoples the world over who have in the past been the focus of research. The purpose of this paper is to give a voice to the quiet minority, those who have done little to contribute towards anthropogenic global warming, those who were first affected, who have solutions that saw them through previous ice ages and warming episodes. The quietness of Indigenous and Traditional Peoples on this topic, was not by a lack of trying but is perpetuated through racism, language, and knowledge superiority; this results in vastly decreased opportunities.

## Methods

### Community locations

Due to the high diversity of Indigenous and Traditional Peoples globally, our case study narratives are from a range of communities, with varying political, cultural, and economic influences in their ability to practice and pass down knowledge of their traditional customs. Due to colonial systems in place across the various communities not all case studies reflect what has been happening in relation to ocean spaces but offer examples across a range of significant cultural habitats. The case study locations were influenced by the heritage of our team members and contributors, with 6 located in the northern hemisphere and 3 in the southern hemisphere.

Our paper focuses on traditional (non-western science) methodologies, that align with our own thinking, position, and strategies. The method we used was Yarning Circle, which has been used extensively in the health sector (Dean 2010; Geia et al. 2013; Goulding et al. 2016; Shay 2019; Walker et al. 2014; Yunkaporta and Kirby 2011). Oral pedagogies in the style of storytelling (yarning) is a shared tradition across many Indigenous and Traditional communities globally, with narratives being a major part of knowledge sharing (Bessarab and Ng'andu 2010; Yunkaporta and Kirby 2011). Yarning circles are an important knowledge sharing approach for many communities, the main goal is a safe space for discussions amongst a group of people, that is informally facilitated, the group take it in turns to speak on a particular subject so all voices and positions are heard. For many Indigenous and Traditional People this is a familiar style of conversation, it allows for relationship building and knowledge sharing in a respectful manner.

The style of yarning that occurred over the Future Seas Workshop Week (Nov 2019), was a combination of collaborative and research topic yarns as described in Bessarab and Ng'andu (2010). The sessions included topics related to each of the Future Seas 2030 Project ‘key challenges’ such as: Blue Economy (Bax et al. 2021, Novaglio et al. 2021, both this issue), Pollution (Willis et al. 2021 this issue), Governance (Haas et al. 2021, this issue), Food Security (Farmery et al. 2020 this issue), and climate-driven species distribution (Melbourne-Thomas et al. 2020, this issue) and more. These sessions were facilitated by co-author Tero Mustonen. The yarning sessions consisted of Indigenous and Traditional Peoples and non-Indigenous Peoples, depending on the topic being discussed. The paper direction was informed by our own diverse and rich collective knowledge and experience, combined with some of these discussions and the case study narratives.

## Case study narratives

**Fig. 4:**
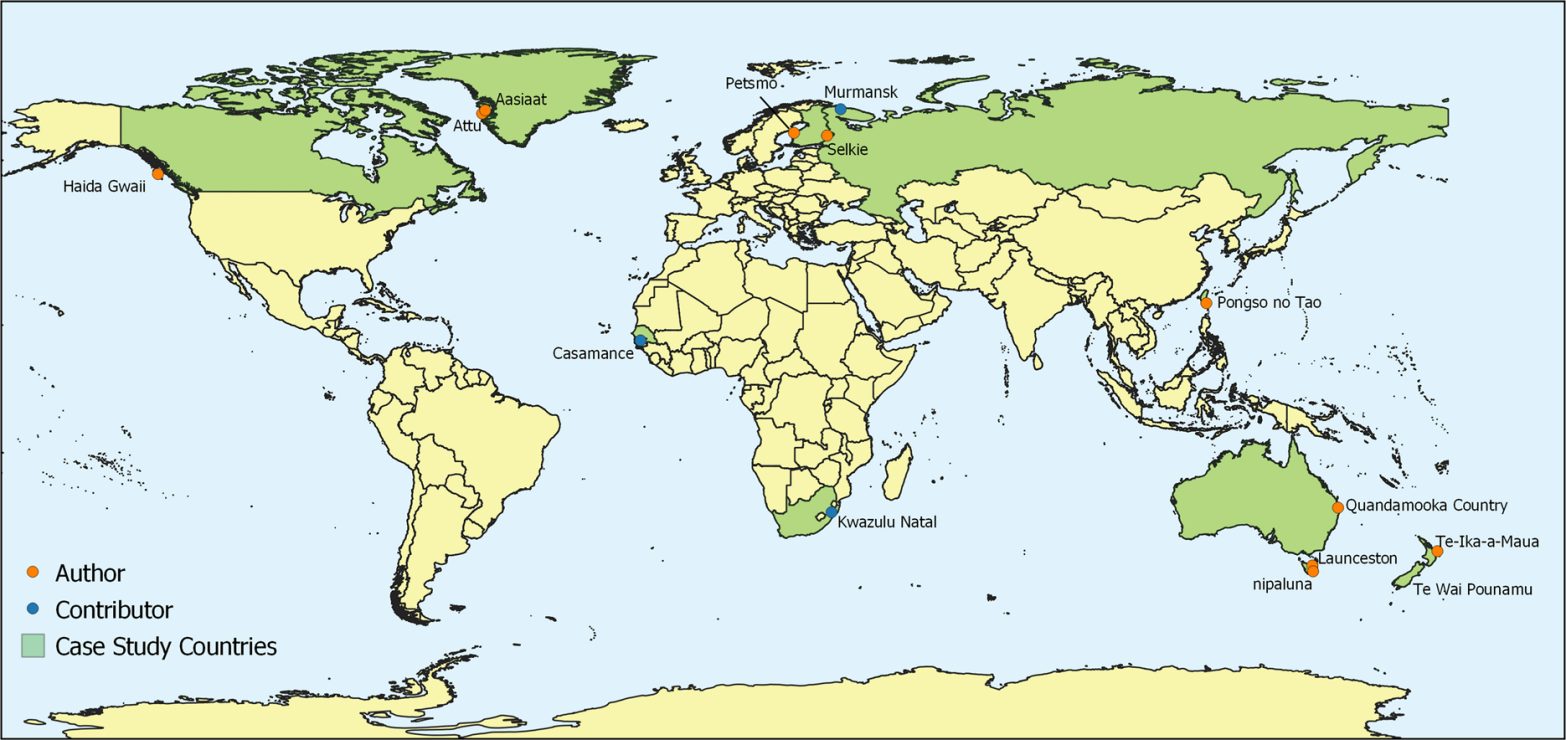
Map of Case Studies featured. Author connections are represented in orange, with case study contributors represented in blue

In order to appreciate the varied knowledge that Indigenous and Traditional Peoples have worldwide we will journey around the globe and hear from communities tied to our team members (see Fig. 4) about how we have come to the current state (Business as Usual), and highlighting where changes are necessary and where activities should be continually supported to achieve future aspirations. Each of the case studies is written by the Indigenous and Traditional authors, giving Indigenous and Traditional Peoples an opportunity to share their diverse experiences. This diversity is reflected in the narratives with variations between the case studies i.e. species names.

We start our journey in the North-Western Pacific Ocean.

### Haida Gwaii (Canada), contributed via hereditary Chief Nang Jingwas (Russ Jones)

Haida Gwaii or “Islands of the People”, is an archipelago lying on the edge of the continental shelf off the north coast of British Columbia (BC) and is the home of the Indigenous Haida Nation. The Haida Nation has about 5000 citizens^2^ and about half currently live in Haida Gwaii. Beginning in the late 1800s, the Haida territory, economy and self-governance was usurped by colonial systems such as Indian Reserves and discriminatory regulations. Haida people approved a Constitution and established an elected Council of the Haida Nation in the early 1980s to represent all people of Haida ancestry. Negotiation of modern treaties and agreements are slowly proceeding, supported by recent Canadian reconciliation approaches such as recognition of rights (Jones et al. 2021a, b). In the Haida case, negotiating interim agreements and management plans for land and marine spaces was catalysed by a mix of Haida political actions and litigation including a court case to prove title to Haida Gwaii that was launched in 2002. The Haida Nation worked with the federal and provincial governments and other coastal Indigenous Nations on integrated ocean management plans^3^ and currently co-manages several large protected areas with marine components.

The Haida Gwaii Marine Plan, endorsed by the Haida Nation and Province of BC, is an example of this collaborative work. It guides marine activities and outlines a future scenario for Haida Gwaii that focuses on a conservation and local economy path:

Twenty years from now Haida Gwaii has followed a path that prioritizes culture, healthy intact ecosystems and sustainable communities. Marine use and development are balanced with high environmental protection standards and a comprehensive network of marine protected areas. Marine industries generally have low environmental impacts and are consistent with the distinct Islands lifestyle. Community growth is based on a diversity of activities that tap into a growing global demand for sustainable seafood and a unique visitor experience (Haida Nation and Province of BC 2015: 32–33).

The Marine Plan identifies about 20% of Haida ocean territory as candidates for marine protection. External drivers and pressures such as climate change and global markets are expected to have a significant impact on the future of Haida Gwaii. Internal drivers include out-migration of youth and its negative effects on community infrastructure such as schools, health care services and transportation. Potential economic opportunities include shellfish aquaculture, increased local benefits from commercial and recreational fisheries, marine-based tourism, and renewable energy development such as wind or tidal power. Fisheries was not fully addressed in the Marine Plan since it lies outside Provincial jurisdiction. The Marine Plan includes detailed objectives and strategies that align with the future scenario. The plan will soon have been implemented for 5 years and is making significant progress.

In general, governance structures for Haida Gwaii plans are based on consensus decision-making. Similar collaborative governance structures are being applied at the Large Ocean Management Area scale for initiatives such as Marine Protected Area (MPA) network planning and shipping and marine protection in partnership with Canada and nearby Indigenous Nations (Jones et al. 2021a). Haida ethics and values (Jones et al. 2010; Jones & Williams-Davidson 2000) and insights from traditional knowledge are incorporated into marine and protected area management plans. Collaborative planning and management are meaningful steps towards reconciliation of Haida and State responsibility which is continuing through negotiation as well as litigation.

Next, we travel up through the Bering Strait into the Arctic and down to Baffin Bay, Greenland.

### Attu, Greenland, contributed via Per Ole (Nuunoq) Frederiksen and Halfdan Pedersen

Attu is north of the Arctic Circle, situated on Greenland’s west coast (see Fig. 4). Over several thousand years, different Inuit-related groups have inhabited Greenland with the present population largely descending from a North American immigration a little over 1000 years ago. A Norsemen group also entered southern Greenland then and stayed until the mid-fifteenth century. A Danish-Norwegian pastor started a Christian mission in 1721 and this is the time referred to as the beginning of the colonial period. In 1774, Denmark closed Greenland off by establishing KGH, Den Kongelige Grønlandske Handel, subsequently prohibiting any development of the people and country. A Danish commercial post was established to the south of Attu in 1759, later called "Illuerunnerit", Gamle Egedesminde, for targeting local species. However, the Danes there died of hunger and disease in the terribly cold winters and the post was moved north of Attu to Aasiaat in 1763. In 1818 Attu was established as a commercial post.

Unknown to many Greenland and Danish people, in the latter half of the 1940s the UN demanded Denmark develop Greenland and to lift the development ban which she did in 1950 (Olesen 2019). During the 1950s and 60 s many habitations in the broader Attu area were compulsorily abandoned and most of the people moved to Attu itself. In the 60 s, Attu people harvested fish alongside the Danes and living from hunting almost became a historic relic. Danish regulatory and societal structure was exported to Greenland and imposed by short-term contracted Danes. Greenlanders had no participation nor authority in their own community (Pedersen 2019, personal communication, 13 November).

In 1964, the Danish government proposed a ban on the use of the Greenlandic language. The bill was renounced after 3 years due to immense opposition and this instigated the mission to achieve self-governance, the Greenlandic People call themselves Kalaallit and their name for their country today is Kalaallit Nunaat. Home Rule was introduced in 1979 to recognise that “the permanent residents in Greenland have fundamental rights to Greenland’s natural resources” (Danish Government 1978) and Greenland’s self-government was introduced in 2009 (Danish Government 2009). However, Denmark continues not to recognise the Kalaallit people’s rights to, or knowledge of, their natural resources. In the 1960s, the Attu population increased from about 50 to 400 and remained stable until 1990. At this time the Greenlandic government promoted education and training, and the Attu population fell to c.200, due to a lack of jobs for highly educated people. This also affected the number of full-time fishers and hunters, and now there are only about 30 full-time fishers and hunters left in Attu.

Locals have in-depth knowledge of Greenland’s climate, such as atmosphere and water temperatures, ice melts, and effects on fishing and hunting resources. The Attu elders recollect ancestral stories handed down from one generation to the next. For example, every 50 summers and winters or so Greenland became warmer or colder. They noticed that the inland icecap kept a steady position until the beginning of the 1500’s. From then, the melting of the icecap quickened, and they noticed during their summer reindeer harvesting the first mountain peaks protruding through the icecap and lakes near the icecap had grown bigger and some had become part of the sea, for example Tasiusarsuaq cove in the Naternaq area (Nuunoq 2019, personal communication, 13 November).

The post-1950 impact of Denmark control on Greenland is described as a technocratic tyranny, which is leading to a cultural genocide. Scientific reports and local observations are inconsistent, resulting in hunting regulations which are also inconsistent with climate change effects on species availability. Greenland halibut and narwhal Total Allowable Catch (TAC) limits suggest the species are rapidly declining, whereas small-scale fishers are harvesting the TAC in unprecedentedly quick times. The regulations are effectively imposing bans on hunting and fishing practices. For example, due to earlier river melts, Arctic char are migrating to sea and fattening earlier (from April to July/August), where the harvest season doesn’t open until June 15 before closing on August 15 (Nuunoq 2019, personal communication, 13 November). This endangers winter supply harvesting, as does the walrus season which ends when the walrus arrives in the Ittoqqortoormiit (Scoresby Sound area) in east Greenland; and the thick-billed murre bird hunting season (Nuunoq 2019, personal communication, 13 November), which now begins after the bird has left the area due to climate change (Merkel et al. 2016).

People have lived in the Attu area a long time prior to colonization because here there is an abundance of seals, walruses, belugas and narwhals, Arctic char, birds, and halibut, among other things, up to this very day. In 2030 the Greenland community wants to put local food products first before resorting to importing food, with local arrangements developed to facilitate this lifestyle, based on local conditions, know-how, mentality, history, and knowledge. They expect their knowledge to be acknowledged by others, regardless of position, rank, creed, colour, ethnicity, and nationality. Attu people will continue to invite scientists to work with them to create regulations, based on their mutual knowledge.

Next, we move southeast through the North Sea and into the Baltic.

### Coastal and Forest Finnish Communities, contributed by Tero Mustonen, Kaisu Mustonen and Eero Murtomäki

The Baltic Sea, today the world's most polluted inland sea, is home of many Traditional and Indigenous cultures. Whilst the Sámi are internationally best known as the Indigenous peoples of the Nordic space, the small nations of the Baltic and coastal communities have preserved unique non-Indo-European relations and cultures with their sea areas until today (Tunon 2018). Some of them, such as Livonians and Izors, also have domestic status of Indigenous peoples.

The UN Convention on Biological Diversity’s Ecologically and Biologically Significant Marine Areas (EBSAs) lists nine important sub-areas of the Baltic. Five of them are discussed here from the point of Traditional and Indigenous relations with the sea (West Estonian Archipelago, Åland Sea and Archipelago Sea of Finland, Kvarken Archipelago, Eastern Gulf of Finland and Northern Bothnian Bay) (EBSA 2018).

Many coastal Finnish and Swedish communities maintain old ways of maritime coastal cultures, for example in the Kvarken-High Coast World Heritage Area of glacial rebound (Mustonen and Mäkinen 2004; Tunon 2018). The island of Maa-Kalla has full autonomy and its own customary laws, even to this day, separate from the laws of Finland and other European countries (EBSA 2018).

Maa-Kalla is a small island in the northern Baltic that arose from the sea due to glacial rebound some centuries ago. It was used as a seasonal base of Baltic herring fishery by the coastal Finnish fishers and family. In 1771 the Swedish King (ruler of Finland at the time) provided the island with full autonomy so that fishermen themselves can rule and decide on all issues of the island. This continues to this day (EBSA 2018) so that Maa-Kalla island is technically owned by the state of Finland but the state has no power—only the traditional customary ruling body consisting of the fishing families. Maa-Kalla is therefore a rare fully autonomous space outside the EU and national legislation built on traditional governance and seasonal occupancy of the island.

The Baltic coastal Indigenous and Traditional communities have also been influenced by the large geopolitical and socio-historical events of the past 100–200 years, most significantly by the Second World War and subsequent modernisation, where the sea was a crucial operational theatre for participating naval powers. Subsequently the Baltic was part of the Iron Curtain separating the Soviet-controlled states from the Neutral countries (Sweden, Finland) and North Atlantic Treaty Organisation (NATO) states (Germany, Denmark). This has made its specific footprint and marking on how Traditional and Indigenous knowledge has survived or been lost, preserved and/or is being revitalized.

In some parts of the Baltic, we need to acknowledge that the traditional maritime culture has been lost, probably permanently, due to the forced relocations of the Soviet era, modernisation and other reasons. In such cases, the examples from the documented practices and cultures may serve as important and stimulating points for present-day action and cultural heritage. Elias Raussi (1966), a tradesman from the community of Virolahti, in Bay of Finland, witnessed fishing families self-organising each year out on the islands for the herring harvests in the early 1800s. This endemic fishery (Mustonen 2014) and locations of harvests were decided by consensus and on a rotational basis so that no one person or family could dominate the harvest locations and each community had a chance to equally participate in the harvest of the fish in their turn in their customary governance.

Revitalising governance of the Baltic to recognize community rights and participation is still relevant today despite its increased role as an oil and cargo transportation route, especially from Russia, out to the global markets. The ethics of the traditional systems, such as the Merikarvia and Pori as well as Kvarken region communities (Mustonen and Mäkinen 2004) could be included in the co-governance of marine protected areas and cultural heritage plans of the Baltic. Equity and justice issues are still very relevant in the context of the Izhorian plight of the oil terminals and environmental pollution of the Baltic on the Russian sector (EBSA 2018). This small nation could still be supported to preserve its homeland and unique way of life. Under Russian legislation for example the concept of traditional land (and marine) use [The law on Territories of Traditional Nature Use TTNU, Зaкoн o тeppитopияx тpaдициoннoгo пpиpoдoпoльзoвaния (TTHУ)] could be implemented to preserve the Izhorian coastline and community rights.

Another region-wide equity issue pertains to the rights of the small-scale fishers across the Baltic, where the prevention of access to quotas and inability of young fishers to enter into the trade is being challenged by the present-day governance of the Baltic, favouring large trawling fleets and industrial practices. This also means that traditional knowledge will be discontinued if there are no new fishers.

All across the Baltic, including traditional knowledge in community-based monitoring and baselines, especially regarding the eutrophication and other pollution events on the coasts and at sea, could be a measure that would reform and improve the work of the Baltic Marine Environment Protection Commission (HELCOM, https://helcom.fi), the state-driven international cooperation on the environmental protection of the Baltic (EBSA 2018; Tero Mustonen et al. 2018). The UN-led EBSA work to identify crucial marine areas of the Baltic, recently identified locations and steps to include traditional knowledge and community-based monitoring into their processes (EBSA 2018).

We now head over land southeast to arrive on an island in Taiwan.

### Pongso no Tao, Taiwan, contributed via Sutej Hugu

We call ourselves ‘Tao,’ with a population of about 4300 people. Pongso no Tao (literally meaning the ‘Homeland of Tao people’) is a small northern volcanic outlier of the Batanes Islands southeast of Taiwan (now labelled ‘Lanyu’ or ‘Orchid Island’ on the official atlas). There are six independent tribal communities, each with origin myths and legendary stories of their own. Nurtured by the richness of the Large Marine Ecosystem of the Kuroshio Ocean Current, the Tao people have lived ‘the original affluent society’ with their comprehensive traditional ecological knowledge and practices for millennia. These non-hierarchical and unspecialized egalitarian tribal communities are without chiefs or ruling elders, but functional leaders responsible for various production, construction, and ceremony activities or events, with only a complementary sexual division of labour within households. Tao people follow their unique time reckoning system called ‘ahephep no tao’ (evening of people), which is an original eco-calendar to keep track of both monthly lunar cycles and the annual solar cycle.

Pongso no Tao has been colonized by numerous foreign powers since the 19th century, most recently the Chinese Nationalist government which attempted to assimilate the Tao people into their culture (Arrigo et al. 2002; Hugu 2012). Despite numerous attempts in recent history to assimilate the community, the Tao people have remained strong although their shift from a system that was once that of a vigorous and optimized Indigenous marine culture to one of unnatural poverty has had numerous impacts on the Tao people (Arrigo et al. 2002).

Since 2010 we have started to build three knowledge bases. The first is Tao Cultural Digital Archive, second is the Pongso no Tao Tribal Geospatial Information Systems Database and third is the Tao People Ethnobiology Knowledge Base (Hugu 2012). In conjunction with the three knowledge bases we have established Tribal Heritage Keepers Groups for elders from each patriarchal lineage to join families from the six tribes on the island. As an alternative to modern capitalist development and an extractive economy, these groups provide guidelines of tribal governance for island management for future generations (Hugu 2012, personal communication, 12 November).

Following the eco-calendar along with its ecological and phenological knowledge contents there are three major ceremonies to initiate each season with critical ethic value. One of the ceremonies is mivanoa for rayon season, all men, young and old, should gather on the community beach to have a ritual of summoning the flying fish school back, and reconfirming their inter-species pact between the flying fishes and Tao people from the ancient time, to implement the rights of nature and order of the living world. From the mythology of the Tao people, that was the ancestor of the noblest black-winged flying fishes teaching the ancestors of the Tao people how to appropriately harvest and treat the flying fishes for survival of both species. In the same story, there is the first account of the arrangement of works and ceremonies all around the year for Tao people (Hugu 2012, personal communication, 12 November).

In the Tao marine governance institution, rayon season is for the fishing of migratory species only. Fishing on coral reefs is completely prohibited during this period. Fishing of flying fish is stopped when they come into the peak time for reproduction. Whilst in the other seasons the coral reef fishery is opened. They are however divided into three categories of oyod (good), rahet (bad) and jingangana (not-for-eating), this is to spread and mitigate the pressure on the food chain. Oyod fish are for women and children whilst rahet is for men and elders only. Less valued species are considered rahet and only for men, with some species in rahet labelled kakanen no rarakeh, which is only for elders (Hugu 2012, personal communication, 14 November).

The Tao worldview is expected to explicitly and implicitly guide game rules for daily life and the principles for social cultural praxis.

We then travel further south into the Pacific Ocean.

### Ōpōtiki, Te-Ika-a-Maui—Te Ika a Maui me Te Wai Pounamu (New Zealand), contributed by Kimberley Maxwell

New Zealand (NZ) is a group of islands in the South Pacific Ocean-Te Moana-nui-a-Kiwa. Polynesian ancestors migrated to these islands onboard multiple waka (canoes) from across the Pacific from c.1200AD (Hogg et al. 2003). Those ancestors brought with them their knowledge, skills and culture, and then adapted it to better suit the sub-tropical to sub-Antarctic NZ context. NZ Māori is the collective term for the Indigenous members of the 100 + iwi (tribes) and 800 + hapū (sub-tribes) of NZ (Te Puni Kokiri (Ministry of Maori Development), 2018). “Indigenous” is a descriptor used by most Māori and Pasifika scholars to position themselves within the postcolonial era (Smith et al. 2016).

From the late 1700s European whalers, sealers, missionaries, and settlers began to colonise NZ. In 1840, more than 500 Māori leaders signed the Treaty of Waitangi with British Crown representatives, which initiated the formation of the NZ government (Kaiser et al. 2019). The principles and history of the Treaty of Waitangi are fundamental to understanding NZ’s Māori-Crown relationships.

During the time of writing this contribution I am working on behalf of one of my Iwi, Te Whakatōhea, who are located around Ōpōtiki township, in the eastern Bay of Plenty, North Island (Te-Ika-a-Maui), NZ. Therefore, I share the Whakatōhea context in relation to the blue economy. There are multiple (± 6) hapū (sub-tribes) associated with Te Whakatōhea. Whakatōhea were a prospering nation at the turn of the 18th century. During colonisation my ancestor and Whakatōhea chief, Mokomoko, was wrongfully imprisoned and hanged for the murder of Reverend Carl Volkner, the fertile lands of our tribe were confiscated, and we were ordered onto a reservation in the corner of our traditional territory. As a child, I bore witness to the Government pardon of Mokomoko, and as an adult I witnessed the Mokomoko pardon being passed into law. The wrongful imprisonment and murder had dire consequences on our people’s socio-economic wellbeing ever since. However, Whakatōhea have remained culturally strong within their relatively ecologically healthy territories and are in the 20 + year process of settling our grievances with the Crown, while simultaneously planning for a prosperous future.

As an Iwi we have a vision to ‘be the food basket of the world’ in the broadest sense of the meaning—to nourish people’s physical, spiritual, and mental well-being, now and into the future. This vision is based on a Whakatōhea narrative about our ancestor, Tāpuikakahu, who uttered the words, ‘te kai hoki i Waiaua.^4^’ The Waiaua River is one of our ancestral rivers and it has long been an important food basket for Whakatōhea. Mārearea (*Galaxiid* spp.), eels, flounder, tītīko (mudsnails), pipi (*Paphies* spp.) and other resources are bountiful in the Waiaua estuary. Our customary fisheries, commercial offshore aquaculture venture and kaitiakitanga (reciprocal relationship of care between ourselves and the environment), are an important part of achieving this vision. Although our lands were taken, Whakatōhea are developing a marine and coastal area plan to characterise and define how we will manage activities in this part of our territory, in collaboration with external entities, including our tribal neighbours.

We will also need to mitigate the risks of climate change, such as increased marine heatwaves on our rohe moana (marine territory) while maintaining cultural integrity. Marine heatwaves have the potential to stress our shellfish species, and potentially reduce recruitment. Our harvesting activities need to be adjusted to address the impacts of these major stressors, for example, by changing the mussel catch limits and by growing mussels deeper in the water column if the water is cooler there.

At the end of 2019, Whakaari (White Island) erupted during a tourist excursion to the island (Graham-McLay 2019). Many lives were lost and the eastern Bay of Plenty tribes, including Whakatōhea, placed a rāhui (temporary prohibition of take) on the sea and coast south-west of the Whakaari Island for over two weeks out of respect (Te Runanga o Ngati Awa, 2019). This halted mussel harvesting on the farm, which was due to take place in the area, and all coastal activities except for the search and rescue. This rāhui was adhered to and reflects the importance of Māori cultural practices above economic risks, better known as cultural bottom lines. This scenario highlighted the need for Iwi to discuss their priorities relating to practicing values in the marine space.

This case study demonstrates how cultural practices can be continued regardless of whether they are legislated or not if the community continues to practice and honour them.

A short trip across the Tasman Sea and we reach the island continent of Australia.

### Lutruwita & Quandamooka Country, Australia, contributed via Dean Greeno, Jamie Graham-Blair and Mibu Fischer

According to the Australian Bureau of Meteorology, there are eight major climate zones across Australia (https://www.abcb.gov.au/Resources/Tools-Calculators/Climate-Zone-Map-Australia-Wide), ranging across those zones are hundreds of First Nations Australians (http://nationalunitygovernment.org/pdf/aboriginal-australia-map.pdf). With this geographical variance in climate, so too are there variances amongst the nations in lores, beliefs and management of the lands, waterways, and skies. This makes for one complex system for the present-day colonial government to understand. Lutruwita sits in a globally recognised hot spot for climate change (Pecl et al. 2019), the impacts that the Palawa People are seeing around their coasts is shifting fast and concerns many of the old people.

Gathering shells was a fun activity because the beach was an extension of our backyard and playground, and we were attracted by their colour and shape. As kids it was part of a natural activity as we ran along with Mum though, as I came to learn, it was also an introduction to an important part of our culture (Greeno 2014).

The importance of connection to country is reflected by renowned artist and Palawa Truwana Elder Aunty Lola Greeno in the statement above. Her experience represents the intrinsic nature of the relationship that many First Nations Australians have with country. It also shows how her deep understanding of the maireener shells^5^ has allowed her to notice changes in their abundance. What is causing these changes is unknown at present by the local community, but they speculate it is linked with climate change. The importance that maireener shells have to the Palawa community is immense, the contemporary use of marine shell necklaces goes beyond economic purposes, the shells are a link to culture.

Gathering shells is an important practice that is continued on Minjerribah (North Stradbroke Island, QLD), however these shells are for food. Eugaries (*Plebidonax deltoides*) are a significant cultural resource and food staple combined with other shellfish species for the Quandamooka People, with the entire coastline of Minjerribah and Moorgumpin (Moreton Island, QLD) once covered in towering middens^6^ (Cope 2020; Hall 1984; Hall & Bowen 1989; Moore 2011). Connections to marine species and habitats are important sources of nutritional, emotional, spiritual, and cultural health for coastal and seagoing First Nations Australians. Quandamooka People were recognised as being the Traditional Custodians of their lands and sea in 2011 by the Federal Government after a 16 year battle ("Delaney on behalf of the Quandamooka People v State of Queensland," [2011]). Part of this agreement has meant that management of some lands and coastal waters is back in the hands of Traditional Custodians. As a result the Quandamooka People have numerous Traditional Custodian Rangers, who work on country to conserve, maintain and connect to the land and sea (Fischer et al. 2019).

Quandamooka People have been caring for country for over 21,000 years. There are traditional narratives about life before the last sea level rise 8000 years ago (Lee et al. 2009; Machado, 2014; Stephens and Sharp 2009). These types of narratives are not unique to Quandamooka, there are traditional narratives, lores and beliefs tied to landscapes that were lost by the rising tides around Australia (Nunn and Reid 2015). These histories tell stories of human survival through climate events, which can only suggest that there is much to be learnt from First Nations Australians about adapting to a changing climate.

## Additional Indigenous observations and experiences

In addition to the case studies from the authors communities the following stories have been shared from colleagues of the SnowChange Cooperative in Finland, recorded for the purpose of the Future Seas 2030 process, and consented by the communities to share here.

### Sámi, Russia, shared by Pauliina Feodoroff, Tero Mustonen, Kaisu Mustonen

#### Maritime Skolt Sámi Diaspora and Nexus of arctic geopolitics

Rybachy Peninsula is in Murmansk region, Russia. It is the ancestral homeland of the Skolt Sámi people, an Eastern Sámi language group of Indigenous peoples in the European North. Rock art from the area are linked with Sámi occupancy (Shumkin 2000). Rybachy Peninsula is *Kikker’njarg* in Skolt Sámi, referring to the three-cornered bone located in the head of a reindeer that is said to be of similar shape as the Peninsula itself (Tanner 1929).

Skolt Sámi preserved their endemic Indigenous governance of village council, *Sida såbbar* the longest, until 1944. Then they were relocated to present-day Finland, leaving their marine fishing areas and coastal occupancy of the Rybachy Peninsula to Soviet Union that annexed the area.

Petsamo region where Rybachy Peninsula is located had been already partially modernized and occupied by the Finnish state in 1920s when the Peace Treaty of Dorpat provided the young northern European country access and ownership to this traditional Skolt Sámi area (Tanner 1929). The Finns who arrived occupied Sámi dwelling and fishing sites and introduced large-scale fishing fleets to the region. Subsequently also the industrial mining of ores, especially nickel started in Petsamo, forcing many Sámi to relocate internally and suffer the loss of reindeer pastures, sacred landscapes and alterations to nomadic routes (Mustonen and Mustonen 2013).

Between 1917 and 1944 Rybachy Peninsula was divided between Finland and the Soviet Union. On the Soviet side the Stalin purges caused severe impacts, for example the Snaulin family was executed (Stepanenko 2002) and many ethnically Finnish and Sámi families put to labour camps and interned in the centre of the Murmansk region, Lovozero. During WW2 the peninsula saw heavy fighting between the Germans and Soviets. In between the Skolt Sámi lost their pasture lands, fishing areas and home areas. We can call this the Skolt Sámi Maritime Diaspora that continues to this day. Rybachy Peninsula is a closed-off military zone in the Russian Federation.

The Skolt Sámi have retained a significant amount of maritime knowledge and deep connections to the Rybachy Peninsula even today. The present generation of Skolt Sámi leaders is actively documenting oral histories and knowledge regarding their past presence in the area. Documentation of place names from Rybachy Peninsula, such as *Cabb’njargg*, describing rich areas of seastars on a cape; *Ainne’suolla*, a place associated with female Gray seals (*Halichoerus grypys*), as well as very white snow; *Soti’suelo*, an island where you needed to melt the snow for freshwater (from Tanner 1929), and place names of the region associating with the culturally-relevant Greenland Shark (*Somnious microcephalus*) indicate the rich endemic knowledge the Rybachy Peninsula Sámi developed over the centuries regarding their ecosystems. This wealth of knowledge embedded in the toponyms is an important ecological source of information for developing the territory in future.

### Zulu Nation, South Africa, shared by Sabelo Mzileni and Thomas Nkunat

Traditional Zulu fishing communities of the province of Kwazulu Natal strive for a return of traditional governance of their marine and freshwater areas, feeling that the present-day management is not respecting their rights and the current urgencies. To them a solution would be the re-establishment of traditional community-based governance:

*Our parents and even we have witnessed these climate changes and seen the negative impact on the Lake. In the face of a range of climate changes that result in limited fishing days due to severe storms and unusually high winds, drought in some places, unseasonal flooding, rising waters and the disappearance of certain previously common species upon which we relied on for food, the fishers are finding it difficult to rely on traditional knowledge. This is made worse by the fact that the Government has introduced stringent criteria for the allocation of fishing rights and many youth are excluded on the grounds that they do not have ten years of experience. This then makes it difficult for the fishers to share their Indigenous knowledge with the next generation.*

*The introduction of the extractive ‘Ocean Economy’ policy, known as* Operation Phakisa *in South Africa has further exacerbated this situation. The state is introducing off-shore oil and gas and mining and industrial style aquaculture projects. Many of our fishers feel trapped in a fast-changing environment and unable to impart the wisdom of the Elders onto the youth of the future generation. We are deeply concerned about the impact of these developments on the fish and other sea life. In our culture the Elders have knowledge of these resources. We want to once again become the guardians and caretakers of the lake, forest and our lands.*

*In our Zulu culture it is very important for young persons to learn from their elders. We have a lot of traditional rituals and processes to ensure that the young ones learn the practice of* ukulondaloza *(preservation/conservation) and other practices that enable knowledge of how to fish, to use resources from the coastal forest for medicine and traditional healing practices, how to use grass and wood for building and so on are transferred from one generation to another. Our dream is that we can participate in the co-management of our lands, lakes, and forests so that we can ensure that these natural resources are managed sustainably and will be there for our children and future generations.*

(Mzileni and Nkunat 2019).

### Kawawana—Indigenous and community conserved area, Senegal, shared by Salatou Sambou

To realize steps towards renewed self-governance and maintaining of ecological health, Salatou Sambou, a fisherman and an Indigenous leader from Senegal positions their local work into thoughts of a sustainable, reformed future:

*Our Kawawana,*^7^* because of its limited size, cannot offer sustainable long-term solutions alone. Our resources move, they migrate. That’s why we need to strengthen our protection actions and rules in Kawawana, but also engage in fruitful discussions with other communities along the coast. We need more interactions between stakeholders and communities from the district level to the regional level, to create a communal space that will generate significant and concrete results by 2030. Extending our area of influence will secure the resources it provides for all of us. Kawawana is quite small, yet on our scale we can already witness species migrating elsewhere because of climate change. Communities must acknowledge that and be on the same page on these kinds of issues, because if the marine resources disappear, our lives are at risk. Communities must understand that protecting their environment and its resources is also their responsibility, in collaboration with governmental and non-governmental organizations. We need to be all together in this. Communities need to realize that they will be the first to benefit from conservation and restoration measures, even before the government. Sure, there will be national and international political benefits from that, but for local communities it’s a matter of survival. Today, we, on the ground, depend on national and international policies, but local people really need to start thinking about taking care of themselves and retrieving their governance in their everyday lives. When awareness will be raised on this, I think everything can change quickly* (Sambou 2019).

The case studies shared above are only a small example of the many experiences from those communities, and are but a mere drop in the ocean for the global experiences Indigenous and Traditional Peoples are faced with daily, the Preface for this journal edition also highlights additional community experiences (Mustonen et al. 2021, this edition).

## Our reality

The next sections will build off the case studies and yarning session conversations on the current reality for many coastal Indigenous and Traditional Peoples. The case studies have highlighted past, present and (for some), future visions for their communities, from these and the yarning sessions three main themes were identified. For the purpose of the discussion, agency, colonisation, and globalisation are consistent themes throughout the case studies. All are seen as having significant impact towards persistent systems where the communities were unable to continue or adequately continue their traditional cultural practices relating to oceans and coasts. In a colonised and Western dominant system Indigenous and Traditional People’s agency is reduced, and in some cases, non-existent, with colonisation and globalisation structures contributing towards their ability to create change for themselves. We will first start with undesirable business as usual scenarios identified from the narratives, before moving to future scenarios that can be modelled or built upon current practices to effect desired change for a more sustainable 2030 for coastal Indigenous and Traditional Peoples.

### Business as usual—undesirable

Many coastal Indigenous and Traditional Peoples are the first to be directly impacted by climate change (Abate and Kronk 2013). With half the world's population living within 200 km of the coast (Neumann et al. 2015), coastal Indigenous and Traditional People are at risk of a second wave of attempted genocide in the form of climate change and globalisation. However, the ability for many of these communities to enact change is influenced by external factors, commonly enforced through foreign sovereign powers. The case studies reveal what many scholars have identified that colonisation is the structure that underpins many of the current disparities between Indigenous and non-indigenous communities (Griffiths et al. 2016; Paradies 2016; Schultz 2018; tebrakunna country and Lee, 2019; Tuhiwai Smith 2012). The current inequitable nature of laws, access to areas, learning systems and more all stem from genocide, assimilation and many other horrific techniques used to eliminate Indigenous and Traditional Peoples to gain access to territory. In all the case studies, restrictions and removal from territory were key to disconnecting Indigenous and Traditional Peoples from managing ocean resources, which has led to the general Business as Usual approach where Indigenous and Traditional Peoples are not considered when it comes to our oceans.

Highlighting that colonisation is a structure and not an event allows opportunities to understand current undesirable scenarios where Indigenous and Traditional People are forced to work within the constraints of the Western system. The influences of political boundaries, as with the Finnish and Sami examples relating to the influence of displacement.

Despite many governments acknowledging the importance of international declarations and protocols which pertain to Indigenous and Traditional Peoples, such as the UN Declaration on the Rights of Indigenous Peoples (UNDRIP), the implementation of those standards at an internal national level is still seemingly a struggle. Even internal legislation is not often enforced in some places, as is the case with Kalaallit Nunaat (Greenland) and the eternal struggle for the Kalaallit population to contribute to managing their resources. For many community members it directly impacts on their livelihoods and survival in a remote region of the world. Despite government legislation (Danish Government 1978), the Kalaallit aren’t considered to have rights to resources, which goes against what is outlined in UNDRIP Article 32. Article 32 states: Indigenous and Traditional Peoples have the right to determine and develop strategies for the use of their territory and resources; and that states should consult and seek approval for the use of resources, and adequate compensation should be paid (United Nations 2007). Current fisheries management in Greenland is guided by Danish regulations, whose management plans are informed by old and incomplete studies, with no regard for the observations of the Kalaallit community. With rapidly changing conditions from climate change—i.e., ice melts, unprecedented temperature variances and changes to species distribution—the current Business as Usual approach to management unfairly disadvantages the Kalaallit community from being able to continue their fisheries livelihoods, as the management regulations do not align with changes to species movements and ocean conditions (Melbourne-Thomas et al. 2020 this issue).

On top of colonial structures is the constant threat of globalisation and the idea of a one world approach and the losses associated with that, like language, cultural practices, territories, access to resources and livelihoods, this is in addition to the climate change threats that Indigenous and Traditional Peoples face (Dana and Dana 2007). Globalisation is often thought to be linked with Westernisation (Pieterse 1994), and due to this interpretation is often seen as a negative to Indigenous and Traditional Peoples. In many contexts colonisation and westernisation occurred in unison, however this was not the case for all, the people of Pongso no Tao were colonised by Japan and later China. Hirst and Thompson (2019) identified that if globalisation was to continue as it is perceived, it would lead to governance systems that would threaten nation states and that there would be a backlash against globalisation. Unfortunately, the nation states that would be threatened do not relate to the nations of Indigenous and Traditional Peoples. The impacts to these communities from globalisation can be identified from the case study narratives. For the Zulu Nation from South Africa the introduction and expansion of oil, gas and mining imposes further restrictions to fishing zones, impacting on traditional fishers. The same is happening in the Baltic where large fishing fleets are favoured, pushing out traditional subsistence and commercial fishers. New gas pipelines and oil terminals are examples of unstoppable megaprojects affecting small-scale fishers. Not only do these threaten community livelihoods, but in these examples the importance of fishing practices and the passing down of knowledge during the practices has been expressed as something that is inhibiting younger generations from accessing that knowledge (Patrick 2019).

Exclusion is not a new concept for Indigenous and Traditional Peoples with many western management practices focused on zoning restrictions for conservation and restoration purposes (Campbell et al. 2012; Day 2002; Day et al. 2008; Grantham et al. 2013; Halpern et al. 2008; Lunn and Dearden 2006), which inadvertently prohibits Indigenous and Traditional Peoples from accessing livelihood spaces. Management isn’t the only reason for exclusion from space, conflict over territory also shuts communities out of traditional areas, in the Baltic many communities were excluded from their traditional coastal and sea spaces as a result of conflicts between warring nations—i.e. WW2 and then the subsequent Communist rule between 1954 and 1992 on the Eastern Baltic. The resulting exclusion adds to the impacts of colonisation, globalisation and climate change by reducing agency of Indigenous and Traditional Peoples to remove or shift barriers to continuing traditional practices. Agency as we refer to it, is the ability for Indigenous or Traditional Peoples to enact change. There are some communities who have worked through barriers and established management plans in line with cultural beliefs and practices, but fall short when they need to enforce these plans with groups outside of the community. In several of the case studies there are desires by the community to provide information towards management practices, to enable their inclusion in coastal and ocean practices, but also economies. Communities want to gain their rights to self-governance and use traditional management techniques, without limiting their ability to participate in livelihood opportunities. Current benefits to Indigenous and Traditional Peoples from participation in management practices are often measured in terms that are outlined by the western or oppressing system (Austin et al. 2018), and do not take into account the wider benefits the communities are wanting to achieve.

The above outlines some of the undesirable actions, systems and practices that continue to persist in today’s world, and into the future. They are considered undesirable as they continue the narrative of leaving Indigenous and Traditional Peoples out of the story or forcing traditional practices into a western structure. It perpetuates colonial dominance and discrimination. To benefit all of earth’s people, there needs to be equity between all. If the current dominant systems proceed without consideration for Indigenous and Traditional Peoples, then these communities and wider population will continue to be impacted greatly from a Business as Usual approach. The connection with the marine environment for many will be jeopardised for the benefit of those who aim to financially benefit from extractive use of resources.

### A future scenario—more sustainable 2030

The desired future of the Indigenous and Traditional Peoples within our team is one of self-governance, cultural respect and recognition for their continued sustainability efforts and knowledge. A future where traditional knowledge is recognised, and Indigenous and Traditional People can provide a service to the wider community that enables the nurturing and protection of our ancestral lands, seas and skies for future generations. The case studies highlight current approaches, or lack thereof, and identify the scenarios that are deemed undesirable for Indigenous and Traditional Peoples, but what does a more sustainable 2030 look like for these communities?

There are numerous examples where traditional knowledge has been integrated into various development and management arrangements, including in our case studies (Dam Lam et al. 2019; Prasetyo et al. 2020; Thompson et al. 2020), whilst this has been a step forward in the inclusion of traditional knowledge, there is growing sentiments that this is not enough. Thompson et al. (2020) found that when Indigenous peoples are in leadership positions, the outcomes are very different than when they are managed by an external group. There is a push away from integration, towards co-management and indigenous-led approaches (Fisher and Parsons 2020). These are steps in the direction of sovereignty and self-governance over natural resources, and further examples can be seen in the Taiwan and Finnish case studies, Pongso no Tao and Maa-Kalla respectively, who are governed by traditional laws. To allow this to happen power is needing to be forfeited and handed back to communities. The Pongso no Tao community are exemplifying this through their return to a traditional way of life, utilising modern technologies to assist in the rebuilding of their society. This frames the importance on knowledge and passing that knowledge on to future generations (Fernández-Llamazares and Cabeza 2018; Magni 2017; Nalau et al. 2018; Opare 2016). They have also made a point to avoid capitalist development and extractive economies as it conflicts with the Tao worldview.

Raising awareness of disadvantages, discrimination and marginalisation is not to say that these communities are without positives. From the case study narratives there are multiple examples of what more appropriate inclusion of Indigenous and Traditional Peoples can look like. The diversity amongst colonisers and the variance in internal structures that have been put in place to reduce the agency of the colonised, all influences the ability for different Indigenous and Traditional Peoples’ communities to continue their guardianship over their ancestral territories.

Similar to Thompson et al. (2020) review on Indigenous participation, a survey conducted by Hedge et al. (2020) shows that representation of Indigenous peoples within marine science research (at least in Australia), was mainly in regards to data collection. The consistent othering of traditional knowledge is what has continued the tension between the two worldviews (Fig. 2). The differences between the Indigenous and Traditional Peoples case studies within this paper, is an example of how academics could shift their thinking solely on traditional knowledge and understand the benefits in including an indigenous methodology or worldview when conducting future marine and coastal research and monitoring and making management decisions. Key lessons from the Haida Gwaii experience to improve appropriate participation in management include the value of cooperative visioning and planning of ocean spaces and equitable co-governance arrangements that share power and responsibility (Jones et al. 2021a). It is one example of co-produced management framework using traditional knowledge and western science. The broader issue of Haida rights to the land and sea is playing out in a lengthy legal battle for recognition of Haida title. Where agreement between Indigenous peoples and the State on land and ocean use is not possible, incremental progress may be possible through mechanisms such as the designation of land or marine territories as Indigenous Community Conservation Areas (ICCAs) while building on international commitments to establish protected areas (Aichi Target 11). In Canada, the Haida Gwaii Marine Plan is an example of the colonised working within the colonisers’ systems to create change and influence using their traditional customs.

Globalisation also can contribute positively to the narrative, as it has allowed the Indigenous and Traditional Peoples collectively to come together and increase our voices on issues that are having direct impacts to our quality of life (Dana and Dana 2007). An increase in the use of modern technologies, including social media, has enabled many communities to not only connect with each other, but engage with western practitioners who are wanting to make a difference to the livelihoods of these communities (Berg-Nordlie 2018). The sharing of different avenues of managing coloniser governance systems is creating a more western educated platform for communities to advocate for social, cultural, political, and educational changes.

All case studies show marginalised and minority groups, who are struggling for cultural survival in the various current systems. All have lost territory or their capacity to decide on the land and sea uses, and few have a clear path forward to achieve equity regarding their marine resources and meaningful decision-making around ocean use. Whilst we have identified here a few examples that can be considered successful, there is still a long way to go for colonial structures in giving up power and allowing communities to self-govern. Moving towards an inclusive system, which encourages decolonial thinking and facilitates Indigenous and Traditional Peoples to successfully achieve self-determination and self-governance practices will support a more sustainable 2030 for all.

### Sharing the Ocean’s wealth

The true extent of knowledge and management practices that is currently known by Indigenous and Traditional Peoples are not known by western science, and it is only when true equality is reached through transmission of power over these resource areas by foreign powers that this breadth in knowledge will be shown. As the race for domination of the marine environment heats up, Indigenous and Traditional Peoples want to make sure their customary marine areas are recognised, and they are considered as more than stakeholders in the process. The idea of staking a claim in the evolving marine realm is not a new one with numerous communities interested in participating and leading pathways back to a more traditional way of managing ocean and coastal areas (*The Sea Within: Marine tenure and cosmopolitan debates* 2017). However, the extractive consumerist lifestyle of the western worldviews, compared to the holistic based Indigenous worldviews is of concern to Indigenous and Traditional Peoples who fear their knowledge and culture will be westernised through incorrect interpretation of practices resulting in the misuse of traditional knowledge, which only perpetuates the colonial system that exists in today’s world. Avoidance of this can occur through Indigenous and Traditional Peoples leadership and adequate co-management principles in place (White 2020).

If we take a literal understanding of wealth from oceans, then the plethora of economic benefits from ocean and coastal resources are not adequately shared with coastal Indigenous and Traditional Peoples (Spalding et al. 2016). Even so, wealth to these communities (and some western communities) extends beyond those of an economic nature and relate to the spiritual and cultural affiliations with these species and habitats. Due to conflicting resource use, historically Indigenous and Traditional Peoples’ values is considered of low importance. It is this disadvantage that needs to be addressed, to enable fair and equitable access and say over ocean and coastal resources. One solution may be enacting seascape use and occupation studies which have happened for example, in Inuit areas of Canada and the coast of Murmansk, Russia (Tero Mustonen and Mustonen 2013; Riewe 1992). Transfer of previously globally ‘unknown’ uses and customary right areas may, provided the context is there, lead to Indigenous and Traditional Peoples’ maritime rights being recognised.

We are not suggesting here that because historically Indigenous and Traditional Peoples have been marginalised, disenfranchised, and oppressed that all communities perceive themselves as such. There are many communities who are practicing culture and thriving, it’s the western ideals of development that influence ideals around poverty, education, and income. This only highlights the inequalities between the different worldviews and how they contribute towards society, and ultimately towards a sustainable future.

## Fair future

For a fair ocean future for all of earth’s people we will next suggest ways forward that shift the narrative for inclusion of Indigenous and Traditional People in the ocean’s future, towards a future that empowers communities to actively participate in their future. Whilst Indigenous and Traditional Peoples continue to face barriers, educating allies is essential in pushing for our inclusion. Moving forward toward a more desirable future, would see a shift in allies leading, to one where they start to actively ask themselves questions—Am I the best person to be leading this? Is there an Indigenous or Traditional person who can lead this? Do I really understand this situation fully? Who is identifying the benefits? A shift in the way practitioners engage with communities and a push from the bottom-up level as well as governance shifts at the highest levels will work together towards including Indigenous and Traditional Peoples in ocean and coastal spaces.

Our group is not alone in wanting to express the desire and struggles of Indigenous and Traditional Peoples, coastal Indigenous representatives are voicing their perspective to direct how to achieve our desired Future Seas in multiple places at multiple levels (locally, regionally and globally). For example, at the OceanObs’19 Conference held in Hawaii, September 2019, the Indigenous delegation (Aha Honua) presented a Coastal Indigenous Peoples declaration acknowledging our continuous connections and responsibilities to the ocean and coastal systems, and calling on the ocean observing community to formally recognise the Traditional Knowledge of Indigenous Peoples worldwide, and the articles of UNDRIP, and to create meaningful partnerships with Indigenous and Traditional Peoples to advance the UN Sustainable Development Goals and the goals of the UN Decade of Ocean Science for Sustainable Development.

UNDRIP in particular provides norms that guide relationships between Indigenous Peoples and signatory states including Indigenous rights to self-government, control over natural resources, language rights and autonomous legal, cultural and educational institutions (see e.g., Sullivan and Kymlicka 2007: 595) from which injustices can be identified and criteria for reconciliation can be developed (Jones et al. 2021a).

The UN General Assembly declared 2010–2020 the Third Decade for the Eradication of Colonialism. And with colonisation a major theme and threat to the ability of communities to make decisions for themselves about their adaptability for their continued survival from the very real threats of climate change, from displacement due to loss of land, to food resource changes from species range shifts. There are also the unknown global threats that influence the global human population, like the spread of SARS-CoV-2 from Wuhan, China to the rest of the world.

## Recommendations

Whilst there are seemingly many limitations to utilising Indigenous and Traditional Peoples knowledge a few of the more noticeable ones are the prejudice these communities face from western society and the constant need for legitimacy of their knowledge systems. Although beginning to be widely accepted in environmental sciences, traditional knowledge has deep cultural connections and should not be taken out of hands of Indigenous and Traditional Peoples. This has been difficult due to language barriers and systemic oppression and silencing. Whilst there are some scholars who may say there are numerous studies and papers expressing the importance of Indigenous and Traditional Peoples to environmental management, many of these papers are not written by people belonging to these communities. They are often interpreted by well-meaning western trained scholars and practitioners. The time is coming for Indigenous and Traditional Peoples to not only be respected for their knowledge and for an equal seat at the table, but also to be handed over power to be able to make decisions over their coastal and ocean spaces.

As members of Indigenous and Traditional communities we offer five recommendations moving forward and beyond current Business as Usual approaches. These range from actions by individual researchers to systematic changes to address ocean threats and empower Indigenous and Traditional Peoples in ocean planning and management.

- Become more self aware of Indigenous issues, as scientists and practitioners in ocean and coastal science/management, and challenge processes, structures and strategies that do not include Indigenous and Traditional Peoples’ voices.
- Question yourself, your position and your projects, do they require Indigenous and Traditional Peoples engagement and if so, are you the right person to be leading this research question?
- Widen your worldview, our ancestors have not survived previous climate change events without learning and passing down relevant knowledge, it is real knowledge, do not force someone to legitimise it.
- Engage communities in addressing climate change issues in whatever ways they can contribute. Climate change is having and will have real impacts to our communities, which will not only force movements and changes to livelihoods, it will also have drastic influence over entire cultures. The current systems of power are limiting their involvement and change has to start somewhere.
- Actively develop partnerships in integrated ocean management and promote Indigenous and Traditional control and management of MPAs and marine developments. For example, in this era of climate change and adaptation, advancement of Indigenous management of protected areas would be a modest step towards achieving biodiversity targets for protection of marine and coastal areas (Aichi Target 11) whilst empowering local communities and mobilising Indigenous and Traditional Peoples knowledge (see e.g. Ward et al. 2020, this issue).

We feel deeply that Indigenous and Traditional Peoples knowledge is for connecting and living, while western science is for conquering and controlling. There is a need for a paradigm shift and power transition to counter the dominance of industrial civilisation and capitalist globalisation of destruction and corruption. Our contribution is beyond the alternative data and information from our hunting, fishing, and gathering skills. Indigenous and Traditional Peoples knowledge is about our rights and institutions, our knowledge and ethics, the livelihood and wellbeing of our communities and how we are embedded within the ecosystem of life.

The case studies in this paper are remarkable stories of survival against all odds and express the sentiment of people and their communities with powerful determination to rise again—people’s lives are at stake here. Inclusion in the decision-making processes that impact our shared resources is essential to the continued survival of cultures and sustainable oceans. Especially if we are to reach a sustainable future globally. However, this inclusion needs to move from passive reference groups, community meetings and heartfelt statements of wanted change to active participation in management practices, movement towards self-governance of traditional territories, changes to government legislations and international related marine treaties and true reconciliation between all of Earth’s people.

As guardians of our oceans and coastal environments we have an intrinsic connection to the needs of our Mother through our cultural practices and knowledge. We have been able to adapt to her changes over generations, it is important therefore, now more than ever, to listen and include our knowledge if we are to achieve any sort of sustainable future.

## Footnotes

1 Australian Aboriginal war club used for hunting or fighting.

2 All persons of Haida ancestry are defined as citizens according to the Haida Constitution.

3 Protected Area include Gwaii Haanas (“Islands of Beauty”) National Park Reserve, National Marine Conservation Area Reserve and Haida Heritage Site that stretches about 10 miles from shore, SGaan Kinghas (“Supernatural Being Looking Outwards”)—Bowie Seamount Marine Protected Area lies 180 km west of Haida Gwaii, and a further 11 coastal protected areas with a marine component; Integrated marine plans include Pacific North Coast Integrated Management Area and the Marine Plan Partnership; Another initiative underway is designing an MPA network for the Northern Shelf Bioregion.

4 To be the food bowl that feeds the world.

5 Maireener shells is the collective name used to identify shell species used in the cultural practice of shell stringing. Four or five main species are identified including rainbow kelp shells, brown and white rice shells and pink button shells.

6 Accumulations of shellfish, bones and other resources that were used by Aboriginal People, middens are still being added to, to this day. And they also show the composition of diet and provide evidence of continued occupation of an area.

7 Name of the Indigenous and Community Conserved Area.
